# Two New Unspecific Peroxygenases from Heterologous Expression of Fungal Genes in Escherichia coli

**DOI:** 10.1128/AEM.02899-19

**Published:** 2020-03-18

**Authors:** Dolores Linde, Andrés Olmedo, Alejandro González-Benjumea, María Estévez, Chantal Renau-Mínguez, Juan Carro, Elena Fernández-Fueyo, Ana Gutiérrez, Angel T. Martínez

**Affiliations:** a Centro de Investigaciones Biológicas, Consejo Superior de Investigaciones Científicas, Madrid, Spain; b Instituto de Recursos Naturales y Agrobiología de Sevilla, Consejo Superior de Investigaciones Científicas, Seville, Spain; University of Toronto

**Keywords:** unspecific peroxygenase (UPO), gene screening, *Collariella virescens*, *Daldinia caldariorum*, *Escherichia coli* expression, enzyme purification, enzyme characterization, aromatic substrate oxidation, site-directed mutagenesis, active site

## Abstract

Unspecific peroxygenases (UPOs) constitute a new family of fungal heme-thiolate enzymes in which there is high biotechnological interest. Although several thousand genes encoding hypothetical UPO-type proteins have been identified in sequenced fungal genomes and other databases, only a few UPO enzymes have been experimentally characterized to date. Therefore, gene screening and heterologous expression from genetic databases are a priority in the search for *ad hoc* UPOs for oxyfunctionalization reactions of interest. Very recently, Escherichia coli production of a previously described basidiomycete UPO (as a soluble and active enzyme) has been reported. Here, we explored this convenient heterologous expression system to obtain the protein products from available putative UPO genes. In this way, two UPOs from the ascomycetes Collariella virescens (syn., Chaetomium virescens) and Daldinia caldariorum were successfully obtained, purified, and characterized. Comparison of their kinetic constants for oxidation of model substrates revealed 10- to 20-fold-higher catalytic efficiency of the latter enzyme in oxidizing simple aromatic compounds (such as veratryl alcohol, naphthalene, and benzyl alcohol). Homology molecular models of these enzymes showed three conserved and two differing residues in the distal side of the heme (the latter representing two different positions of a phenylalanine residue). Interestingly, replacement of the C. virescens UPO Phe88 by the homologous residue in the D. caldariorum UPO resulted in an F88L variant with 5- to 21-fold-higher efficiency in oxidizing these aromatic compounds.

**IMPORTANCE** UPOs catalyze regio- and stereoselective oxygenations of both aromatic and aliphatic compounds. Similar reactions were previously described for cytochrome P450 monooxygenases, but UPOs have the noteworthy biotechnological advantage of being stable enzymes requiring only H_2_O_2_ to be activated. Both characteristics are related to the extracellular nature of UPOs as secreted proteins. In the present study, the limited repertoire of UPO enzymes available for organic synthesis and other applications is expanded with the description of two new ascomycete UPOs obtained by Escherichia coli expression of the corresponding genes as soluble and active enzymes. Moreover, directed mutagenesis in E. coli, together with enzyme molecular modeling, provided relevant structure-function information on aromatic substrate oxidation by these two new biocatalysts.

## Abstract (precis)

UPOs catalyze regio- and stereoselective oxygenations of both aromatic and aliphatic compounds. Similar reactions were previously described for cytochrome P450 monooxygenases, but UPOs have the noteworthy biotechnological advantage of being stable enzymes requiring only H_2_O_2_ to be activated. Both characteristics are related to the extracellular nature of UPOs as secreted proteins. In the present study, the limited repertoire of UPO enzymes available for organic synthesis and other applications is expanded with the description of two new ascomycete UPOs obtained by Escherichia coli expression of the corresponding genes as soluble and active enzymes. Moreover, directed mutagenesis in E. coli, together with enzyme molecular modeling, provided relevant structure-function information on aromatic substrate oxidation by these two new biocatalysts.

## INTRODUCTION

**FIG 1:**
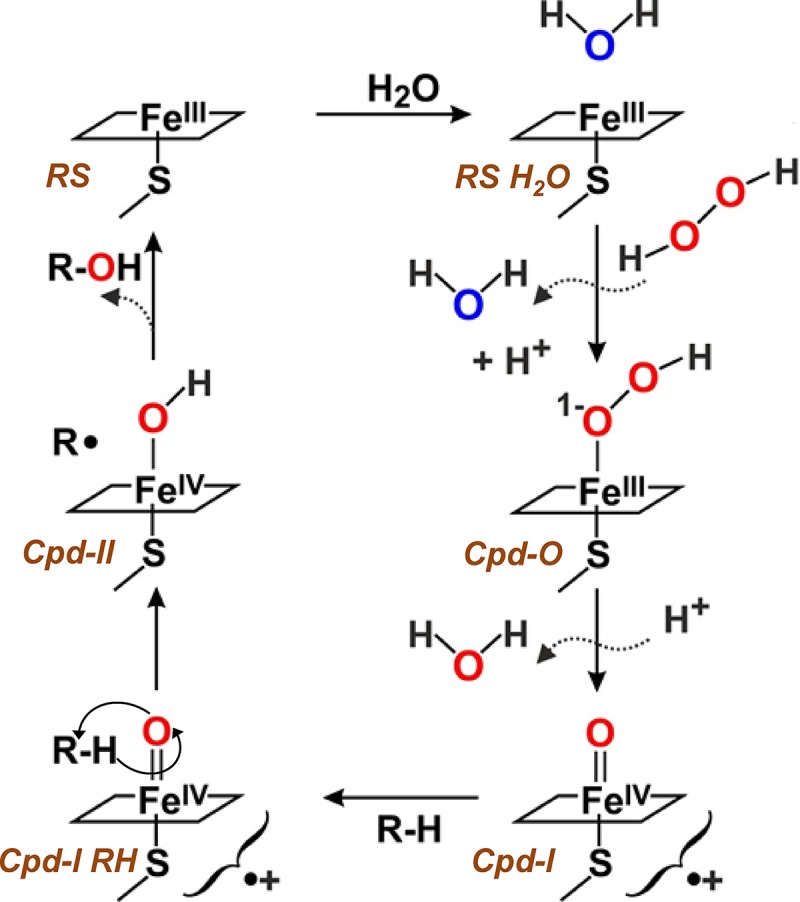
Scheme of the monooxygenase cycle of UPO, including resting state (RS) (Fe^III^ upper coordination position occupied by a water molecule), compound 0 (Cpd-0) (Fe^III^-hydroperoxo complex), compound I (Cpd-I) (Fe^IV^-oxo/porphyrin cation radical complex), and compound II (Cpd-II) (Fe^IV^-hydroxo complex), resulting in substrate (R-H) conversion into hydroxylated product (R-OH) at the expense of hydrogen peroxide being reduced to water (with its second oxygen atom being transferred to the substrate) (adapted from reference 1).

Selective oxyfunctionalization of nonactivated carbon-hydrogen bonds is among the most sought-after reactions in organic synthesis. Unspecific peroxygenases (UPOs) from the heme-thiolate peroxidase (HTP) protein superfamily (1) are ideal catalysts for these challenging reactions, given their ability to catalyze regio- and stereoselective hydroxylations, epoxidations, and other oxygenations that are difficult (and sometimes impossible) to be achieved by only chemical means (2). Additional UPO advantages are their stability and self-sufficiency, two characteristics related to their extracellular nature as secreted fungal proteins. Thus, UPOs are directly activated by hydrogen peroxide, as illustrated by the catalytic cycle shown in Fig. 1, resulting in substrate hydroxylation, and they remain stable under different environmental conditions.

Several thousand UPO-type sequences have been identified in sequenced genomes and genetic databases, corresponding to a variety of species from nearly 20 classes of ascomycetes and basidiomycetes and a dozen fungus-like oomycetes (belonging to the Stramenopiles), which most probably acquired them by horizontal gene transfer from Eumycota (3). Recently, it has been suggested that the number of typical UPOs in fungal genomes could be reduced (to only 113 sequences) by using more-specific search criteria (4), but such estimation seems too conservative.

In any case, it is surprising that UPO proteins of only a few fungal species have been purified and characterized to date: (i) from cultures of the basidiomycetes Agrocybe aegerita (5), Coprinellus radians (6), Marasmius rotula (7), and Marasmius wettsteinii (8) and the ascomycete Chaetomium globosum (9), together with the classical chloroperoxidase (CPO) of the ascomycete Leptoxyphium fumago (syn., Caldariomyces fumago) (10), which belongs to the same protein family, or (ii) after heterologous expression of Coprinopsis cinerea (11) and Humicola insolens (9) genes in Aspergillus oryzae, of an M. rotula gene in Escherichia coli (12), and of an evolved A. aegerita gene in Saccharomyces cerevisiae (13) and Pichia pastoris (14). In addition to the above-mentioned species, UPO activity has been detected in Agaricus bisporus, Agrocybe alnetorum, Agrocybe chaxingu, Agrocybe parasitica, Auricularia auricula-judae, Coprinopsis verticillata, and Mycena galopus (3), but the corresponding enzymes were not purified or characterized.

In the present study, we add two new fungal enzymes, from the ascomycetes Collariella virescens (syn., Chaetomium virescens) and Daldinia caldariorum, to the repertoire of purified and characterized UPOs for reactions of interest. For this purpose, several UPO-type sequences available from databases and other sources (15) were optimized for E. coli expression by the same approach used for producing and engineering (12, 16) the UPO previously isolated from M. rotula (7). The resulting soluble recombinant enzymes were purified, their kinetic constants for veratryl and benzyl alcohols, naphthalene, and 2,2′-azino-bis(3-ethylbenzothiazoline-6-sulfonic acid) (ABTS) oxidation were determined, and their ability to oxygenate unsaturated fatty acids was confirmed. The severalfold-lower catalytic efficiency of the C. virescens UPO than of the D. caldariorum UPO in oxidizing aromatic substrates could be rationalized by site-directed mutagenesis of active-site residues in the former enzyme.

## RESULTS

### Production of new UPOs.

After unsuccessful attempts at *in vitro* activation of UPOs expressed as bacterial inclusion bodies, we focused on their heterologous production in E. coli as active enzymes. For this purpose, the sequences of putative UPO-encoding genes from eight fungi and fungus-like organisms of different taxonomic groups were synthesized and transformed in E. coli to be obtained as soluble and active enzymes. In short, the procedure used included (i) codon optimization from fungal protein sequences, (ii) an autoinduction growth medium, resulting in continuous release of the expression inducer after glucose exhaustion, and (iii) a low incubation temperature (see Materials and Methods for details). In this way, correctly folded heme-thiolate proteins, as revealed by the 450-nm maximum of their CO complexes, could be obtained from the C. virescens and D. caldariorum sequences (the nucleotide sequences used for E. coli production are provided in Fig. S1 in the supplemental material, and the corresponding amino acid sequences are included in Fig. S2 in the supplemental material). These enzymes were subsequently purified and characterized, as described below.

### Purification of the C. virescens and D. caldariorum UPOs.

A common purification protocol was initially applied for isolation of the two recombinant UPOs from E. coli cultures (transformed with genes cloned in the pET23 plasmid), consisting of ion-exchange chromatography and size exclusion chromatography (SEC) after lysis of cells. The lysate from E. coli cultures transformed with the pET23-*Cvi*UPO plasmid included a large variety of proteins of different molecular masses, as shown by sodium dodecyl sulfate-polyacrylamide gel electrophoresis (SDS-PAGE) (see Fig. S3A, lane 1, in the supplemental material). However, a single cation-exchange chromatographic step yielded a sole retained peak with high absorbance at 420 nm, indicative of a heme protein (see Fig. S4A in the supplemental material). This peak included only minor contaminating proteins, as shown by SDS-PAGE (Fig. S3A, lane 2). Purification can be further improved, when required, by SEC, enabling separation (Fig. S4B) of two minor shoulders (fractions I and II) on a main peak (fraction III) of high electrophoretic homogeneity (Fig. S3A, lane 5), which was used for the subsequent biochemical characterization.

However, a similar purification strategy was unsuccessful for isolating the D. caldariorum UPO from contaminating proteins in the cells transformed with the pET23-*Dca*UPO plasmid. Therefore, the gene was cloned in pET28, and purification could be completed by taking advantage of an introduced poly-His tail. The initial metal ion affinity step (Fig. S4C) drastically reduced the very high protein diversity in the E. coli lysate (Fig. S3B, lane 1), with a sole protein being predominant in SDS-PAGE (Fig. S3B, lane 2). When required, a final SEC step (Fig. S4D) enabled removal of several shoulders (fractions I, II, and IV) on a main peak (fraction III), which was used for biochemical characterization given its high electrophoretic homogeneity (Fig. S3B, lane 5). The activity of the D. caldariorum UPO was not affected by the presence of the poly-His tail used for enzyme purification, as shown by thrombin treatment of a UPO sample with the same specific activity before (4.63 U/mg) and after (4.64 U/mg) tail removal.

A summary of the two purification processes is provided in Table 1. The yields of fully purified recombinant UPOs of C. virescens and D. caldariorum after the final Superdex 75 step are about 1.0 and 0.4 mg of pure protein per liter of E. coli culture, respectively. This increases to 7.0 and 2.8 mg per liter of culture, respectively, when the ultrafiltered highly enriched fractions obtained after the first purification step (consisting of cation-exchange and metal affinity chromatographic steps, respectively) are considered. The final specific activities, measured with ABTS as the substrate, were ∼38 U mg^−1^ for the C. virescens UPO and ∼8 U mg^−1^ for the D. caldariorum UPO, with 27% and 13% yields and purification factors of 221 and 125, respectively. The yields and purification factors measured with veratryl alcohol (3,4-dimethoxybenzyl alcohol) were often higher, although the specific activities were significantly lower. It is worth mentioning that activities at the purification process were measured under pH and substrate concentration conditions that are not the optimal conditions determined later with the purified enzymes, as described below.

**TABLE 1:** Summary of the purification processes for the recombinant C. virescens (r*Cvi*UPO) and D. caldariorum (r*Dca*UPO) UPOs from E. coli cultures, using ABTS or veratryl alcohol as the substrate^*a*^

UPO and fraction	Vol (ml)	Protein (mg)	Activity (total U)	Sp act (U · mg^−1^)	Yield (%)	Purification (fold)
r*Cvi*UPO^*b*^						
Cell lysate	60	6,650	1,150 (60)	0.17 (0.01)	100	1
Cation exchange	30	59	292 (36)	4.91 (0.61)	25 (60)	28 (67)
Ultrafiltrate	1	56	240 (39)	4.29 (0.69)	21 (64)	25 (76)
Superdex 75	8	8	309 (14)	38.18 (1.70)	27 (23)	221 (189)
r*Dca*UPO^*c*^						
Cell lysate	100	4,230	243 (54)	0.06 (0.01)	100	1
Metal affinity	70	29	131 (41)	4.48 (1.39)	54 (75)	78 (109)
Ultrafiltrate	1	28	167 (36)	5.90 (1.29)	69 (68)	103 (101)
Superdex 75	10	4	31 (7)	7.68 (1.62)	13 (13)	125 (128)

a Activity, yield, and purification were measured with 1 mM ABTS in 0.1 M tartrate (pH 3) in the presence of 1 mM H_2_O_2_ or with 5 mM veratryl alcohol in 0.1 M Tris (pH 7.4); values for veratryl alcohol are shown in parentheses.

b From an 8-liter E. coli culture.

c From a 10-liter E. coli culture.

### Characterization of the new UPOs.

**FIG 2:**
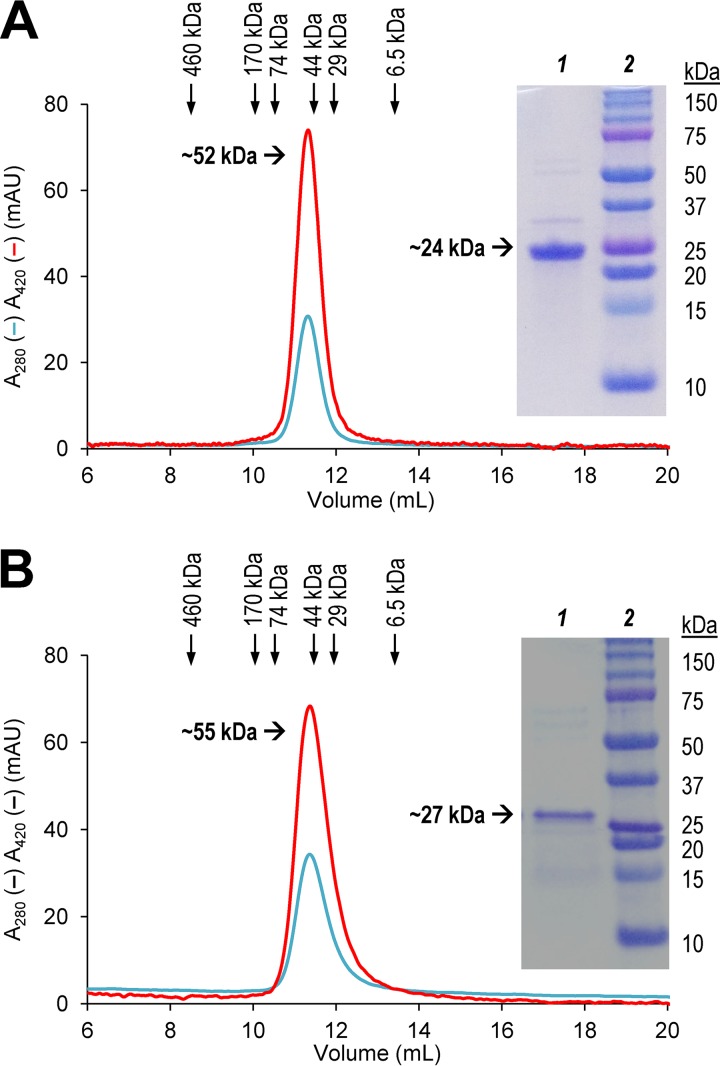
Sepharose 12 chromatography of purified C. virescens UPO (A) and D. caldariorum UPO (B), showing elution profiles at 280 nm (cyan) and 420 nm (red) and elution volumes of the standards (ferritin, aldolase, bovine serum albumin, ovalbumin, carbonic anhydrase, and aprotinin) used for molecular mass estimation (see Fig. S5 in the supplemental material). Insets show SDS-PAGE of the corresponding purified UPOs (lane 1) and standards (lane 2).

The molecular masses of the reduced and denatured UPOs of C. virescens (∼24 kDa) and D. caldariorum (∼27 kDa) estimated by SDS-PAGE together with standard proteins (Fig. 2A and B insets, respectively) were slightly lower than those calculated from their amino acid sequences (29,616 and 29,609 Da, respectively). Moreover, the masses of the native (not unfolded/denatured) UPOs, estimated by Sepharose 12 SEC (Fig. 2) calibrated with selected standards (see Fig. S5 in the supplemental material), corresponded to 52 kDa and 55 kDa (Fig. 2, insets) for the C. virescens and D. caldariorum UPOs, respectively. These results revealed that both UPOs most probably occur as dimers.

The amino acid sequences of the peptides from trypsin hydrolysis of the recombinant C. virescens and D. caldariorum UPOs obtained by nano-scale liquid chromatographic-tandem mass spectrometry (nLC-MS/MS), which covered 80% and 70% of the total protein, respectively (Fig. S2), were identical to the initial UPO sequences used for nucleotide sequence synthesis and optimization. This agreement confirmed that the purified proteins corresponded to the synthesized genes used for E. coli transformation and that no errors were made during the gene synthesis and expression and enzyme purification processes.

**FIG 3:**
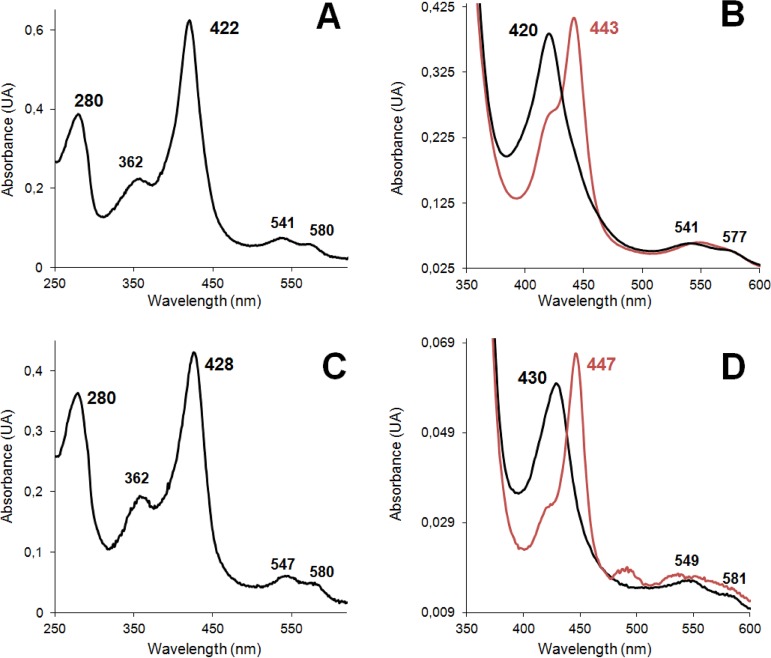
Resting-state (A and C), reduced-state (B and D, black lines), and CO-complex (B and D, red lines) UV-vis spectra of the C. virescens (A and B) and D. caldariorum (C and D) UPOs. See Materials and Methods for details.

Proper folding of and cofactor incorporation into the enzymes produced in E. coli were confirmed by their UV-visible (UV-vis) spectra (at the resting state), showing the strong Soret band at 420 to 430 nm and the two minor peaks at ∼540 and ∼580 nm (Fig. 3A and C). From these spectra, Reinheitszahl values (*A*_420_/*A*_280_ ratios) of 1.6 and 1.3 were obtained for the C. virescens and D. caldariorum UPOs, respectively. Moreover, the presence of a heme-thiolate cofactor (with a cysteine ligand of heme iron) was confirmed by the Soret band displacement to 440 to 450 nm in the spectra of enzyme-CO complexes (Fig. 3B and D), as reported for peroxygenases and cytochrome P450s (5, 17). The heme content in the purified enzyme samples (∼0.5 mol of heme per mole of protein) was measured with the pyridine hemochrome method and used to calculate the extinction coefficients applied to estimate the concentration of active enzyme in kinetic and other analyses.

### Catalytic properties.

Four usual peroxidase/peroxygenase substrates, i.e., the simple aromatics veratryl alcohol, benzyl alcohol, and naphthalene and the aromatic azo dye ABTS, were used to investigate the kinetic properties of the new C. virescens and D. caldariorum UPOs. For proper comparison, the kinetic curves against different substrate concentrations were estimated at optimal pH values determined previously (see Fig. S6 in the supplemental material). Veratryl alcohol oxidation by the C. virescens UPO (Fig. S7A), naphthalene oxidation by both enzymes (Fig. S7B and F), and ABTS oxidation by the D. caldariorum UPO (Fig. S7G) exhibit Michaelis-Menten saturation kinetics. However, oxidation of ABTS by the C. virescens UPO (Fig. S7C) and veratryl (Fig. S7E) and benzyl (Fig. S7H) alcohols by the D. caldariorum UPO shows product/substrate inhibition to different extents, while benzyl alcohol oxidation by the C. virescens UPO (Fig. S7D) exhibits sigmoidal kinetics (fitted to the Hill equation).

From the above-described kinetic curves, the appropriate equations (shown in Materials and Methods) were used to calculate the turnover numbers (*k*_cat_), Michaelis constants (*K_m_*), and catalytic efficiencies (*k*_cat_/*K_m_*) of the two enzymes oxidizing each of the four substrates, together with the inhibition constant (*K_i_*) and Hill coefficient (*n_H_*) values, when applicable (Table 2). Inhibition of the D. caldariorum UPO by veratryl alcohol occurs at high substrate concentrations, with a *K_i_* >280-fold higher than the *K_m_*, while the *K_i_* values were only ∼50 and ∼30-fold higher than the *K_m_* values for benzyl alcohol oxidation by the D. caldariorum UPO and ABTS oxidation by the C. virescens UPO, respectively. Finally, the positive cooperative degree (*n_H_*) in the sigmoidal kinetics observed for the C. virescens UPO revealed that binding of one benzyl alcohol molecule improves the enzyme interaction with additional substrate molecules.

**TABLE 2:** Kinetic constants for veratryl and benzyl alcohol, naphthalene, and ABTS oxidation by the C. virescens and D. caldariorum UPOs^*a*^

Substrate and enzyme	*k*_cat_ (s^−1^)^*b*^	*K_m_* (μM)	*k*_cat_/*K_m_* (s^−1^ mM^−1^)	*K_i_* (μM)	*n_H_*
Veratryl alcohol					
r*Cvi*UPO	2.24 ± 0.03	2,940 ± 160	0.75 ± 0.03		
r*Dca*UPO	2.39 ± 0.09	160 ± 22	14.8 ± 2.1	45,400 ± 7,000	
Benzyl alcohol					
r*Cvi*UPO	62.9 ± 2.3	7,100 ± 600	8.9 ± 0.81		1.6 ± 0.2
r*Dca*UPO	16.09 ± 1.19	179 ± 36	90 ± 19	9,280 ± 2,270	
Naphthalene					
r*Cvi*UPO	1.08 ± 0.07	450 ± 70	2.42 ± 0.40		
r*Dca*UPO	22.1 ± 1.92	332 ± 81	66.8 ± 17.3		
ABTS					
r*Cvi*UPO	157.2 ± 2.6	239 ± 8	656 ± 24.5	7,860 ± 680	
r*Dca*UPO	6.73 ± 0.14	59 ± 6	114.4 ± 10.5		

a Assays were in 0.1 M acetate (veratryl alcohol and ABTS), 0.1 M Tris (benzyl alcohol), or 0.1 M tartrate (naphthalene) at the optimal pH for each enzyme and substrate (shown in Fig. S6 in the supplemental material) in the presence of 1 mM H_2_O_2_. Values are means and standard deviations.

b Calculated as the number of substrate molecules oxidized by one cofactor molecule per second.

Regarding catalytic efficiency, apparent affinity, and maximal turnover values (Table 2), both similarities and differences between the two UPOs were observed. First, ABTS appears to be the best substrate for both of them, as shown by catalytic efficiency, which is notably high for the C. virescens UPO due to its high turnover number (*k*_cat_ of ∼160 s^−1^). Concerning the simple aromatic substrates assayed (veratryl and benzyl alcohols and naphthalene), it is interesting that all of them were better oxidized by the D. caldariorum UPO. This is due to the extremely low apparent affinity of the C. virescens UPO for both veratryl (with a *K_m_* of >2,900 μM) and benzyl (with a *K_m_* of >7000 μM) alcohols and its very low turnover number with naphthalene (*k*_cat_ of ∼1 s^−1^).

**FIG 4:**
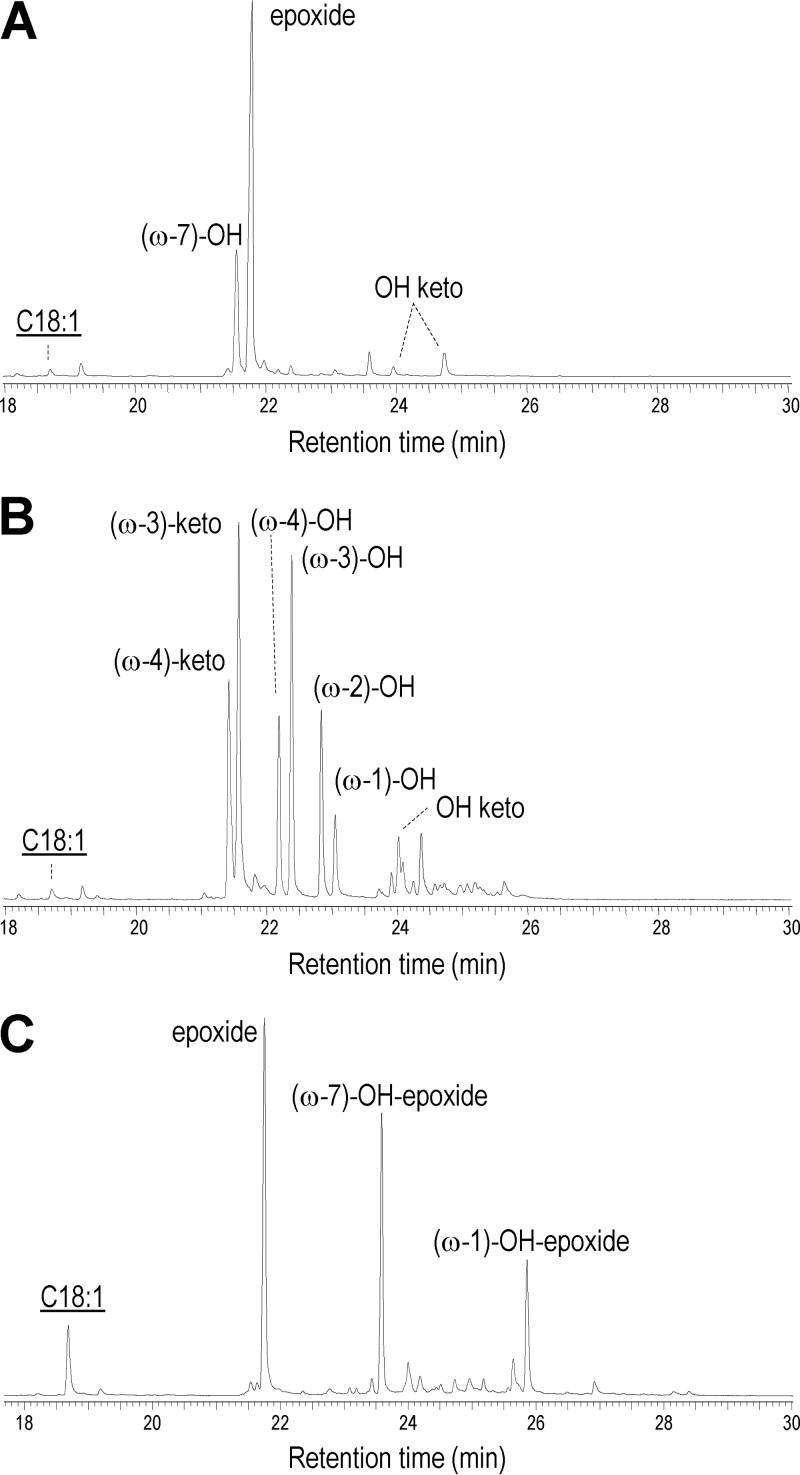
(A and B) GC-MS analysis of reactions of oleic acid (C_18:1_, underlined) with the C. virescens (A) and D. caldariorum (B) UPOs. (C) Previously reported results with recombinant M. rotula UPO (12), shown for comparison. See Materials and Methods for details.

Finally, oleic acid was used to explore the fatty acid oxyfunctionalization ability of the two UPOs, analyzed by gas chromatography-mass spectrometry (GC-MS). As shown in Fig. 4A, 9,10-epoxyoctadecanoic acid is the main product of the oleic acid reaction with the C. virescens UPO, followed by 11-hydroxyoctadec-9-enoic acid [labeled (ω-7)-OH] and traces of hydroxy/keto derivatives of oleic acid. In contrast, no epoxides were found in the reaction with D. caldariorum UPO, which generated only the four 14-hydroxy to 17-hydroxy (ω-4 to ω-1) derivatives and the two 14-keto and 15-keto (ω-4 and ω-3) derivatives of oleic acid, together with a small amount of a hydroxy/keto derivative, as shown in Fig. 4B. For comparative purposes, the results from oleic acid treatment with M. rotula UPO (12), also obtained by heterologous expression in E. coli, are included in Fig. 4C.

### Analysis of site-directed variants at the heme pocket.

**FIG 5:**
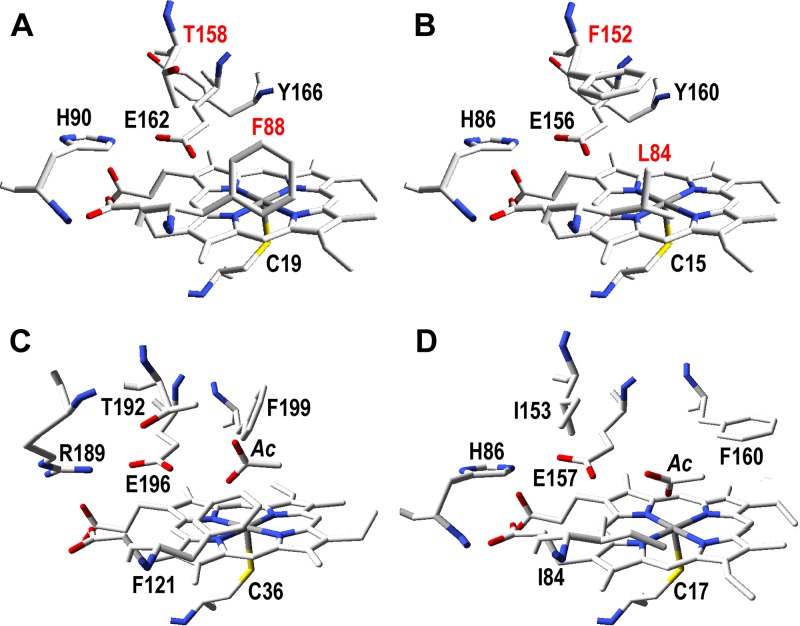
Distal side of the heme pocket in the homology molecular models of the new C. virescens (A) and D. caldariorum (B) UPOs, with indication of the differing homologous residues (red) that were mutated for Table 3, compared with the same region in the crystal structures of the A. aegerita (C) and M. rotula (D) UPOs (PDB entries 2YP1 and 5FUJ, respectively).

Enzyme molecular models were built, using available UPO crystal structures as templates, to explain the very different kinetic constants of the two new UPOs on simple aromatic substrates (and ABTS). Residues at the upper side of the heme pocket (opposite the lower side with a proximal cysteine acting as heme iron ligand) were compared in homology molecular models of the two new UPOs (Fig. 5A and B) and crystal structures of the A. aegerita and M. rotula UPOs (Fig. 5C and D). In addition to the proximal cysteine (Cys19/Cys15), three residues (His90/His86, Glu162/Glu156, and Tyr166/Tyr160) are conserved in the C. virescens/D. caldariorum UPOs. Both enzymes also share the presence of a phenylalanine residue at this side of the heme cavity, being Phe88 in the C. virescens UPO and Phe152 in the D. caldariorum UPO (with Leu84 and Thr158 as their homologous residues in the second enzyme).

To investigate the functional implications of the above-described structural differences in active-site architecture, the F88L and T158F variants of the C. virescens UPO were obtained by site-directed mutagenesis (the double variant could not be expressed as a soluble active enzyme in E. coli) and purified to electrophoretic homogeneity (see Fig. S8 in the supplemental material) by the same protocol used for the wild-type enzymes, showing similar UV-vis spectra (see Fig. S9 in the supplemental material). Their optimal pH values (see Fig. S10 in the supplemental material) and kinetic curves (see Fig. S11 in the supplemental material) for the same aromatic substrates used with the wild-type enzymes were then obtained, and the corresponding kinetic constants were calculated.

Replacing Phe88 in the C. virescens UPO by a leucine residue, as found in the D. caldariorum UPO, strongly increased its catalytic efficiency in oxidizing veratryl alcohol (21-fold), benzyl alcohol (11-fold), and naphthalene (5-fold) (Table 3). These differences are on the same order of those found when the two wild-type enzymes were compared, with the D. caldariorum UPO showing 10- to 30-fold-higher catalytic efficiency in oxidizing these aromatic substrates than the C. virescens UPO (Table 2). In contrast, the F88L variation slightly decreased the efficiency of the C. virescens UPO in oxidizing ABTS, in agreement with the preference for this dye shown by this enzyme compared with the D. caldariorum UPO, which was more evident for the T158F variant described below.

**TABLE 3:** Kinetic constants for veratryl and benzyl alcohol, naphthalene, and ABTS oxidation by the F88L and T158F variants of C. virescens UPO^*a*^

Substrate and variant	*k*_cat_ (s^−1^)	*K_m_* (μM)	*k*_cat_/*K_m_* (s^−1^ mM^−1^)	*K_i_* (μM)
Veratryl alcohol				
F88L	18.6 ± 0.35	1,150 ± 50	16.1 ± 0.31	
T158F	1.56 ± 0.05	3,970 ± 570	0.38 ± 0.05	
Benzyl alcohol				
F88L	63.7 ± 5.8	623 ± 111	102.1 ± 2.8	
T158F	153.8 ± 31.2	1,870 ± 560	82.4 ± 29.3	
Naphthalene				
F88L	2.3 ± 0.05	190 ± 12	12.2 ± 0.63	
T158F	0.17 ± 0.02	255 ± 21	0.68 ± 0.12	
ABTS				
F88L	126.3 ± 11.75	337 ± 56	375 ± 71.9	4,210 ± 1,220
T158F	11.24 ± 0.64	352 ± 61	32.1 ± 4.03	

a Reaction conditions and calculations were as detailed in Table 2 for the wild-type UPOs (at the optimal pH conditions shown in Fig. S10 in the supplemental material).

The improvement in catalytic efficiency on simple aromatic substrates caused by the F88L mutation is due to a significant decrease of the *K_m_*, indicating that in the absence of this phenylalanine residue, a smaller amount of substrate is needed to attain maximal transformation. The additional turnover increase observed for veratryl alcohol oxidation by the F88L variant would be due to better (closer) positioning of the substrate in the Phe88-less variant.

Concerning the T158F mutation, the corresponding variant (Table 3) improved the oxidation only of benzyl alcohol but decreased the oxidation of veratryl alcohol and naphthalene compared with the wild-type enzyme (Table 2). Most probably, the access of these two bulkier aromatic compounds (compared with benzyl alcohol) is hampered by the introduction of a phenylalanine residue at the active site of the C. virescens UPO. However, the effect of introducing a bulky side chain in the T158F mutant was especially evidenced by the 20-fold decrease of catalytic efficiency in oxidizing the large molecule ABTS, in agreement with the lower preference of the D. caldariorum UPO for ABTS than for simple aromatics.

## DISCUSSION

### UPOs are a promising enzyme family to be explored.

The best known peroxide-activated enzymes (peroxidases), such as classic horseradish peroxidase (HRP), prokaryotic cytochrome *c* peroxidase (CcP), and fungal ligninolytic peroxidases (18, 19), belong to the catalase-peroxidase superfamily (20), with the latter two being the first (21) and second/third (22–24) peroxidases with a known molecular structure. In recent years, new peroxide-activated enzymes have been described, including the so-called dye-decolorizing peroxidases (DyPs) in the CDE superfamily and the fungal UPO-type enzymes (including CPO) in the HTP superfamily (25–29). UPOs have been characterized from a variety of fungi, bacteria, and archaea (29, 30), but amazingly, only the structure of the A. aegerita UPO has been published to date (31), together with the classic CPO (32). Thus, the structural-functional information and biotechnological potential of UPOs are strongly limited by the low number of isolated proteins, despite the facts that the first UPO was described 15 years ago (5) and a high number of putative UPO-encoding genes have been identified in sequenced fungal genomes and other databases (3).

The low number of wild (nonrecombinant) UPOs available is due to difficulties in (i) growing higher fungi in pure culture due to complex growth requirements, (ii) detecting in pure culture the UPOs identified in genomes/databases, and (iii) obtaining satisfactory amounts of enzyme. Moreover, the heterologous expression of UPO genes, which should overcome these difficulties, is far from being a straightforward process. Among the host systems used for expressing UPO-encoding genes, production using A. oryzae is a proprietary technology of the company Novozymes (Bagsvaerd, Denmark), and therefore, the strains, vectors, and growth conditions are not publicly available. Yeast expression, as far as reported, requires previous directed evolution introducing mutations that result in structural differences with respect to the wild-type enzymes (33). Overexpression in E. coli as inclusion bodies followed by *in vitro* activation is routinely used for fungal (class II) peroxidases (34–39), but a protocol for *in vitro* activation of UPOs from inclusion bodies has not been developed yet.

### Enlarging UPO diversity.

Fortunately, successful expression of the previously described UPO of M. rotula (7) as a soluble and active recombinant enzyme (r*Mro*UPO) in E. coli has been recently reported (12), covered by a patent application (16). This method was applied here to further explore UPO diversity. In this way, we were able to describe the eighth and ninth UPOs, from C. virescens and D. caldariorum, after those previously described from A. aegerita, C. radians, C. cinerea, C. globosum, H. insolens, M. rotula, and M. wettsteinii (5–9, 11). The original nucleotide sequences of the C. virescens and D. caldariorum UPOs are not available. Only the amino acid sequences were provided by Lund et al. (15), with a putative peroxygenase of Podospora comata (accession no. VBB75842.1) and a hypothetical protein from the sequenced genome (40) of a *Daldinia* species (accession no. OTB17553.1) being the most related sequences in the NCBI database (76 to 77% amino acid identity), respectively. However, optimized nucleotide sequences for E. coli expression as active enzymes could be obtained, and they are now the second and third active recombinant UPOs heterologously expressed in E. coli. Interestingly, their production levels (7 and 3 mg of highly enriched enzyme per liter of bacterial culture, respectively) are higher than that obtained for the M. rotula UPO using the same expression system. However, the heterologous production of UPOs is not a straightforward process, as mentioned above, and only two of the eight UPO sequences assayed could be successfully transformed into soluble and active enzymes in E. coli.

The use of a lactose-containing self-induction medium (41) is an important aspect of E. coli expression of UPOs that, together with the low growth temperature, promotes proper protein folding as an active enzyme. Moreover, since UPOs are fungal proteins, the design and optimization of nucleotide sequences for E. coli expression is a key aspect for their heterologous production. With this aim, the Optimizer software (42) was used, providing both “fully optimized” sequences (i.e., those always using the most frequent codon for each amino acid in the host strain) and “randomly optimized” sequences (alternating several possible codons per amino acid). For UPO expression in E. coli, the first approach was not successful, most probably because of inefficient folding. Random optimization was then screened to identify sequences (see Fig. S1 in the supplemental material), enabling us to obtain soluble and active enzymes. Additional improvements in nucleotide sequence optimization for this E. coli system could further contribute to efficient expression of these (and other) UPO genes, providing an adequate speed balance between protein synthesis and folding (43).

The two new UPOs are ascomycete enzymes to be classified in the group of short UPOs (1), due to their shorter polypeptide chains of 240 to 270 residues (and molecular masses of around 25 kDa). The UPOs from C. virescens and D. caldariorum appear to be dimeric enzymes, as shown by the 2-fold-higher molecular mass estimated by SEC than by SDS-PAGE under reductive conditions. Cys241 of D. caldariorum UPO and one of the C. virescens UPO cysteine residues (Cys178, Cys230, or Cys235) could be involved in dimerization by formation of intermolecular disulfide bridges, as reported for the M. rotula UPO (44) based on its crystal structure (PDB no. 5FUJ and 5FUK). Another dimeric UPO has been reported from M. wettsteinii, based on combined SDS-PAGE and SEC results (8).

### Catalytic properties of the new UPOs.

In general terms, the optimal pH for catalysis by the two new enzymes (pH 3 to 6) is more acidic than reported for the previously characterized UPOs; e.g., pH optima near neutrality have been reported for veratryl alcohol oxidation by the A. aegerita and C. globosum UPOs, as well as for naphthalene oxidation by the latter enzyme and the M. rotula UPO (5, 7, 9). For the two new enzymes, ABTS is also the most efficiently transformed substrate of the A. aegerita and C. globosum UPOs and the second-better substrate of the M. rotula UPO after benzyl alcohol (5, 7, 9). In a similar way, naphthalene is less efficiently oxidized by the two new enzymes, in agreement with results reported for other UPOs (7, 9). Among the two other aromatic compounds, benzyl alcohol is much better oxidized than veratryl alcohol, as also reported for the other characterized UPOs (5, 7, 9), a fact that can be related to its less bulky nature (due to the absence of methoxy substituents), facilitating its access to the buried heme cofactor.

In addition to the above-mentioned aromatics, UPOs are also active on aliphatic substrates. This includes unsaturated fatty acids, as recently shown for the M. rotula UPO (12, 45). In a preliminary analysis, the oxyfunctionalization pattern for the latter enzyme was compared with those obtained for the two new UPOs using oleic acid as the substrate. Interestingly, the three enzymes showed different oxyfunctionalization patterns. While a mixture of epoxide and hydroxy-epoxides (with hydroxy groups at ω-7, ω-1, and other positions) had been reported for the wild (45) and recombinant (12) M. rotula UPO, nearly full conversion with the new UPOs yields as a main product either epoxide by the C. virescens UPO or a variety of subterminal hydroxy/keto derivatives by the D. caldariorum UPO. A complete study of unsaturated fatty acid epoxidation by the C. virescens UPO has been recently reported (54). While epoxides are investigated as reactive molecules for different applications, hydroxy fatty acids are of interest in the manufacture of ester homopolymers of fatty acids.

### Structural-functional aspects of UPO activity on aromatic substrates.

Both the reductive (on H_2_O_2_) and oxidative (on aromatic and aliphatic substrates) UPO half-reactions (Fig. 1, right and left, respectively) take place at the upper side of the heme pocket (1). The main structural difference between the upper heme pockets of the UPOs of C. virescens and D. caldariorum concerns the location of a phenylalanine residue, being Phe88 and Phe158, respectively. A key role of phenylalanine residues at the heme pocket and access channel on UPO activity on aromatic and other substrates has been suggested in the first structural-functional studies on these enzymes, discussed below.

**FIG 6:**
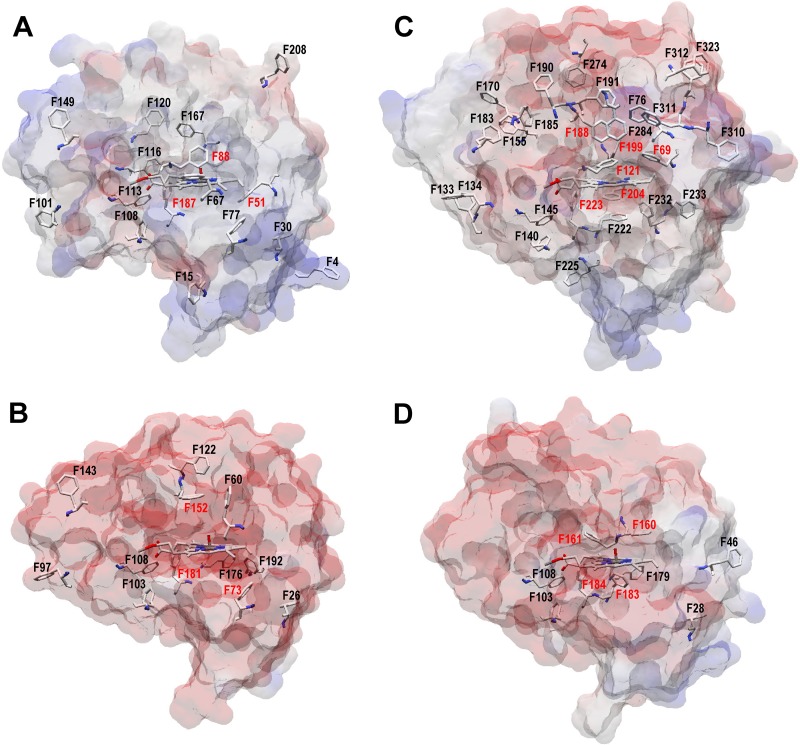
Phenylalanine residues in the C. virescens (A), D. caldariorum (B), A. aegerita (C), and M. rotula (D) UPOs. Semitransparent solvent access surfaces, phenylalanine residues, and heme cofactor (as CPK-colored sticks) are shown. Red labels indicate residues less than 10 Å from the heme iron. Data are from homology models (A and B) and crystal structures (C and D [PDB entries 2YP1 and 5FUJ, respectively]).

The first UPO, isolated from A. aegerita, has up to 27 phenylalanine residues in its mature protein structure (Fig. 6C). The involvement of several heme environment and channel residues (namely, Phe69, Phe76, Phe121, Phe191, and Phe199) in oxidation of polycyclic and other aromatic substrates by this UPO was suggested when its crystal structure was reported for the first time (31). Inversely, the preference of the M. rotula UPO for aliphatic compounds, compared with the A. aegerita UPO preferring aromatics (3, 45–47), could be related to the much lower number of phenylalanine residues at its heme pocket and access channel (only 9 phenylalanines in the whole protein structure) (Fig. 6D). An intermediate number of phenylalanines (17 and 16, respectively) are present in the new C. virescens and D. caldariorum UPOs (Fig. 6A and B, respectively). Interestingly, a docking study of the A. aegerita UPO evolved for heterologous expression in yeast (the so-called PaDa-I variant) identified three of the above-mentioned phenylalanine residues (Phe121, Phe199, and, to a lesser extent, Phe69) as being involved in interaction with all the (aromatic) substrates assayed (33).

As mentioned above, the two new UPOs exhibited differences in the location of an active-site phenylalanine (Fig. 5A and B) and strongly different kinetic constants on aromatics that were investigated by directed mutagenesis of the C. virescens enzyme. Remarkably, replacing Phe88 by a leucine residue, as found in the D. caldariorum UPO, strongly increased the catalytic efficiency of the C. virescens UPO in oxidizing veratryl alcohol and naphthalene (22-fold and 5-fold, respectively), with parallel (8-fold and 2-fold) increases in *k*_cat_, although Phe88 occupies a position equivalent to the above-mentioned Phe121 in the A. aegerita UPO (Fig. 5C). The opposite tendency was observed when Thr158 (similar to Thr192 in the A. aegerita UPO [Fig. 5C]) was replaced in the T158F mutation, which decreases (>20-fold) the UPO catalytic efficiency for the aromatic azo dye ABTS, with a simultaneous decrease of *k*_cat_ (14-fold) and increase of *K_m_* (1.5-fold), with the catalytic activity of the C. virescens UPO again approaching that of the D. caldariorum UPO.

In conclusion, the structural-functional information on the two new UPOs supports the importance of active-site phenylalanines on UPO reactions with aromatic compounds (31, 33). However, it clearly associates the higher activity on simple aromatics in these UPOs with the absence of a phenylalanine residue at the position equivalent to Phe121 in the A. aegerita UPO, while the opposite role has been proposed for this residue in the latter enzyme. In a similar way, the absence of a second phenylalanine in position 158 of the C. virescens UPO (which is also absent in the A. aegerita UPO) confers to the former enzyme its remarkable activity on ABTS compared with that of the D. caldariorum UPO.

## MATERIALS AND METHODS

### Enzyme production.

The protein sequences of eight putative UPOs from the ascomycetes C. virescens, D. caldariorum, and Xylaria polymorpha, the basidiomycetes Laccaria bicolor and Rhodotorula graminis, the mucoromycete Rhizopus delemar, the glomeromycete Rhizophagus irregularis, and the chytrid Spizellomyces punctatus were obtained from NCBI database and reference 15. DNA sequences coding for these proteins were optimized for E. coli expression using the different options provided by the Optimizer software (42), synthesized by ATG:biosynthetics GmbH (Germany), and cloned in the pET23a and His-tag-containing pET28 plasmids under the control of the T7*lac* promoter.

The plasmids were transformed into competent E. coli C41 cells, which were grown for 4 to 5 days in lactose-containing autoinduction medium ZYM-5052 (41) supplemented with hemin at low temperature and agitation (16°C and 180 rpm) to obtain UPOs as soluble and active proteins (12, 16). Cells were harvested (10 min at 8,000 rpm) and suspended in 50 to 70 ml of 10 mM Tris (pH 7.4), 5 mM dithiothreitol, and 20 mM EDTA for cultures with pET23-cloned genes or in 10 mM Tris (pH 8), 0.3 M NaCl, and 20 mM imidazole for cultures with the pET28-cloned genes. Lysozyme (2 mg · ml^−1^), DNase, and sonication were used to lyse the cells, and the soluble fractions containing the recombinant UPOs were recovered after debris removal (45 min at 15,000 rpm followed by 1 h at 36,000 rpm).

As a step before protein purification, an assay to detect the presence/absence of UPO in the E. coli extracts was performed based on the capacity of reduced (ferrous) heme-thiolate proteins to form a complex with CO, which shifts the main peak from 420 nm to 440 to 450 nm, as shown in difference spectra (48). For this purpose, the soluble fractions (200 μl) were mixed with 200 μl of phosphate (pH 8) and adjusted to 1 ml with Milli-Q water, and their spectra (300 to 700 nm) were recorded with a Cary 60 spectrophotometer before (Fe^3+^ resting-state enzyme) and after (Fe^2+^ reduced enzyme) the addition of Na_2_S_2_O_4_, followed by flushing with CO (CO-enzyme complex).

### Enzyme purification.

The C. virescens and D. caldariorum recombinant proteins showed the 440- to 450-nm maximum in the above-described assay and were therefore subjected to further purification using two different approaches, depending on whether the gene was cloned in the pET23a or in the pET28 plasmid.

The C. virescens recombinant enzyme could be successfully purified from the E. coli lysates with the gene cloned in the plasmid without a His tag (pET23) by using two chromatographic steps in an Äkta (GE Healthcare) fast protein liquid chromatography (FPLC) system. Since the C. virescens UPO has a theoretical isoelectric point of 7.7, cation-exchange chromatography was first applied using a HiTrap SPFF column (GE Healthcare, USA) in 10 mM Tris (pH 7.4) to retain positively charged proteins. The retained proteins were eluted with a gradient of the same buffer supplemented with 1 M NaCl and concentrated with an Amicon 3K instrument (Sigma-Aldrich, USA). The second purification step was SEC with a Superdex 75 column (10/300 GL; GE Healthcare, USA) in 10 mM Tris (pH 7.4) with 0.15 M NaCl. Protein elution was followed at 420 nm (heme proteins) and 280 nm (all proteins).

Purification of the D. caldariorum enzyme was facilitated by pET28 cloning, after unsuccessful ion-exchange purification of the protein obtained with the untagged pET23a (used for the C. virescens enzyme). Since pET28 has a His tag, the purification was carried out by immobilized metal ion affinity chromatography with a HiTrap IMAC FF 5-ml column, using the same FPLC system. First, the column was loaded with 0.1 M NiSO_4_ to provide the metal ions where the tail histidines will be bound after sample injection in 10 mM Tris (pH 7.4), 0.3 M NaCl, and 20 mM imidazole. The retained enzyme was eluted with 0.5 M imidazole buffer, using a 0 to 50% gradient at a flow rate of 0.5 ml · min^−1^. The UPO-containing fractions were concentrated in Amicon ultracentrifuge filters and applied to an SEC column (Superdex 75 10/300 GL) using an isocratic flow of 10 mM Tris (pH 7.4) and 0.15 M NaCl at 0.4 ml · min^−1^. As in the previous case, protein elution was followed at 420 and 280 nm. The eventual effect of the poly-His tail on the enzyme activity was evaluated after its removal with thrombin (49).

Purification yield was followed by quantifying proteins at 280 nm (in an Thermo Scientific NanoDrop 2000) and activity by measuring 1 mM ABTS oxidation in 0.1 M tartrate (pH 3) using 1 mM H_2_O_2_ (5 mM veratryl alcohol oxidation in 0.1 M Tris [pH 7.4] was also monitored) along the different purification steps. Enzyme purification was followed by 12% PAGE in the presence of 0.1% SDS and ∼1% mercaptoethanol (present at a 5% concentration in the loading buffer), reducing disulfide bridges, and unfolding proteins (50). The pyridine hemochrome assay was used to assess the concentration of heme in the purified enzymes by adding pyridine to the enzyme reduced with sodium dithionite (51). Molar extinction coefficients were then calculated according to Beer’s law using the above concentration of heme and the enzyme absorbance at 420 nm. The resulting ε_420_ values were 114.2 mM^−1^ · cm^−1^ and 98.3 mM^−1^ · cm^−1^ for the recombinant C. virescens and D. caldariorum UPOs, respectively.

In addition to the above-described SDS-PAGE analyses, the native molecular masses of nondenatured purified UPOs were estimated by SEC in a Sepharose 12 10/300 column (GE Healthcare, USA) using 10 mM Tris (pH 7.4) with 0.15 M NaCl and ferritin (460 kDa), aldolase (170 kDa), bovine serum albumin (73.5 kDa), ovalbumin (44.0 kDa), carbonic anhydrase (29.3 kDa), and aprotinin (6.5 kDa) as molecular mass standards. Proper folding and binding of the cofactor were evaluated by inspecting the UV-vis spectrum of the resting state of the enzymes in 10 mM phosphate (pH 7.4) using a Cary 60 spectrophotometer. Formation of a complex between the chemically reduced enzyme (ferrous form) and CO, characteristic of active heme-thiolate enzymes as mentioned above, was assessed in 0.2 M phosphate (pH 8) after addition of Na_2_S_2_O_4_ and CO flushing.

### Peptide sequencing.

Identification of the purified recombinant UPOs of C. virescens and D. caldariorum was confirmed by nLC-MS/MS analysis of the trypsin-digested peptides (using the Easy-nLC-LTQ Orbitrap Velos equipment) and comparison with the peptides from theoretical trypsin digestion of the C. virescens and D. caldariorum UPOs. N-terminal sequencing was carried out by automatic Edman degradation (52) (using the Procise 494 protein sequencer from Applied Biosystems). These assays were performed at the CIB (CSIC) Proteomics and Genomics Facility (member of the ProteoRed-ISCIII network) and the Protein Chemistry Facility, respectively.

### Molecular modeling and directed mutagenesis.

The molecular structures of the C. virescens and D. caldariorum UPOs were modeled at the Swiss-Model server (https://swissmodel.expasy.org) (53) with the A. aegerita (PDB no. 2YP1) and M. rotula (PDB no. 5FUJ) UPO structures as templates. The F88L and T158F mutations were introduced in the C. virescens UPO gene using the Expand Long Template PCR kit from Roche (Basel, Switzerland). PCRs were run using *ad hoc* oligonucleotides (along with their reverse complementary counterparts) (mutated triplets in bold and substituted nucleotides underlined): (i) F88L mutation, 5′-AAC CGC CAT AAC CTG **TTG** GAA CAT GAT GCA TCT C-3′; and (ii) T158F mutation, 5′-ACT TAC ACC GTT CAG CAG CGT ATC **TTT** AGT TAC GGT GAA ACG-3′.

The PCRs (50-μl volume) were carried out in an Eppendorf (Hamburg, Germany) Mastercycler pro-S using 30 ng of template DNA, 0.5 mM each deoxynucleoside triphosphate (dNTP), 125 ng of direct and reverse primers, 5 units of Expand Long Template PCR system polymerase mix (Roche), and the manufacturer’s buffer. Reaction conditions were as follows: (i) initial denaturation step of 1 min at 95°C; (ii) 22 cycles of 30 s at 95°C, 30 s at 60°C, and 7 min at 68°C; and (iii) final elongation step of 7 min at 68°C. The mutated variants were produced in E. coli and purified as described above for the wild-type enzyme.

### Enzyme kinetics.

Kinetic constants for typical substrates used in previous UPO descriptions (namely, veratryl and benzyl alcohols, naphthalene, and ABTS) (5–9) were estimated after determining the optimal pH for each enzyme and substrate. For this purpose, reactions at saturating concentrations of veratryl and benzyl alcohols (10 mM), naphthalene (20 mM), ABTS (2 mM), and H_2_O_2_ (1 mM) were analyzed in the range of pH 2 to 10, using 0.2 M Britton-Robinson buffer. Formation of the veratraldehyde (ε_310_, 9.30 mM^−1^ · cm^−1^), benzaldehyde (ε_280_, 1.40 mM^−1^ · cm^−1^), α-naphthol (ε_303_, 2.03 M^−1^ · cm^−1^), and ABTS cation radical (ε_436_, 29.30 M^−1^ · cm^−1^) products was followed in a Cary 60 spectrophotometer.

Kinetic curves were obtained at the optimal pH for each enzyme and substrate by varying the concentrations of veratryl alcohol (from 0.07 μM to 4.9 mM, in 0.1 M acetate), benzyl alcohol (from 20 μM to 0.1 M, in 0.1 M Tris), naphthalene (from 15.1 μM to 0.5 mM, in 0.1 M tartrate with 5% ethanol), and ABTS (from 0.07 μM to 4.9 mM, in 0.1 M acetate) in the spectrophotometer cuvette (maintained at 25°C) and adding a final concentration of 1 mM H_2_O_2_ and 20 to 700 nM enzyme. The reactions were triggered by the H_2_O_2_ addition, the time was adjusted to ensure that the reaction rate was in linear phase, and values were calculated as the change in absorbance over time. All reactions were carried out in triplicate.

The plotting and analysis of the above-described curves were done with SigmaPlot 11.0 by nonlinear least-squares fitting the *k*_obs_ values to equation 1 (Michaelis-Menten model), except for (i) veratryl and benzyl alcohol oxidation by D. caldariorum UPO and ABTS oxidation by the C. virescens UPO and the F88L variant of C. virescens UPO, which were adjusted to equation 2 (inhibition model) and (ii) naphthalene oxidation by both UPOs and benzyl alcohol oxidation by the C. virescens UPO, which were adjusted to equation 3 (Hill model):

(1) $$f=\frac{k_{\text{cat}}S}{K_{m}+S}$$

(2) $$f=\frac{k_{\text{cat}}}{1+\frac{K_{m}}{S}+\frac{S}{k_{i}}}$$

(3) $$f=\frac{y_{0}+k_{\text{cat}}S^{n_{H}}}{K_{0.5}{}^{n_{H}}+S^{n_{H}}}$$

where *k*_cat_ is the turnover number (corresponding to the number of substrate molecules that are oxidized by one cofactor molecule per second), *S* the substrate concentration, *K_m_* the Michaelis constant, *K_i_* the inhibition constant, *y*_0_ the *y* axis intercept, *n_H_* the Hill constant, and *K*_0.5_ the substrate concentration for half saturation.

### Fatty acid oxyfunctionalization.

Oleic acid was used to investigate the oxyfunctionalization abilities of the two new UPOs. For this purpose, 30-min reactions were performed with 0.1 mM substrate, 0.8 to 1.4 μM enzyme, and 1.25 to 2.50 mM H_2_O_2_ in 50 mM phosphate (pH 7) containing 20% acetone. Chromatographic analyses were performed with QP2010 Ultra GC-MS equipment, using a fused-silica DB-5HT capillary column from J&W Scientific (USA). The oven was heated from 120°C (1 min) to 300°C (15 min) at 5°C · min^−1^. The injection was performed at 300°C, and the transfer line was kept at 300°C. Compounds were identified by mass fragmentography and by comparison of their mass spectra with those of authentic standards. Quantifications were obtained from total-ion peak areas, using external standard curves and molar response factors of the same or similar compounds.

### Data availability.

All data are open-access available in the main article and its supplemental material.

## Supplementary Material

Supplemental file 1
