# Supplementary material for: Two New Unspecific Peroxygenases from Heterologous Expression of Fungal Genes in Escherichia coli
Source: Appl Environ Microbiol. 2020 Mar 18;86(7):e02899-19. doi: 10.1128/AEM.02899-19 (PMC7082571; doi:10.1128/AEM.02899-19)
Supplement: Supplemental file 1 [file AEM.02899-19-s0001.pdf]

**SUPPLEMENTAL MATERIAL**

# Two New Unspecific Peroxygenases from Heterologous Expression of Fungal Genes in *Escherichia coli*

**Dolores Linde,[a] Andrés Olmedo,[b] Alejandro González-Benjumea,[b] María Estévez,[a] Chantal Renau-Mínguez,[a] Juan Carro,[a] Elena Fernández-Fueyo, [a+] Ana Gutiérrez,[b] Angel T. Martínez [a#]**

[a]Centro de Investigaciones Biológicas, CSIC, Ramiro de Maeztu 9, E-28040 Madrid, Spain
[b]Instituto de Recursos Naturales y Agrobiología, CSIC, Reina Mercedes 10, E-41012 Sevilla, Spain
[+]Present Address: Technical University of Delft, The Netherlands
[#]Corresponding author (ATMartinez@cib.csic.es)

Supplemental material includes optimized nucleotide sequences (**Fig. S1**) and amino-acid sequences (**Fig. S2**) of the *C. virescens* and *D. caldariorum* UPOs, SDS-PAGE during UPO purification (**Fig. S3**), main steps in UPO purification (**Fig. S4**), calibration of Sepharose 12 column (**Fig. S5**), pH profiles of wild-type UPOs (**Fig. S6**), kinetic curves of wild-type UPOs (**Fig. S7**), SDS-PAGE of the purified UPOs and variants (**Fig. S8**), UV-vis spectra of UPO variants (**Fig. S9**), pH profiles of UPO variants (**Fig. S10**) and kinetic curves of UPO variants (**Fig. S11**).

## A

```
ATGGAACTGGACTTTAGTAAATGGAAGACCCGTCAGCCGGGCGAATTCCG   50
TGCCCCGTGCCCGGCTATGAATTCTCTGGCCAACCACGGTTTTATCCCGC  100
GCGATGGCCGTAATATTACCGTAGCCATGCTGGTTCCGGTCCTGCAGGAG  150
GTCTTCCACCTGTCCCCAGAGCTGGCGCAGACGATCTCTACTCTGGGTCT  200
GTTTACCGCTCAAGACCCTTCCAAAGGCGTATTCACTCTAGACGACCTGA  250
ACCGCCATAACCTGTTTGAACATGATGCATCTCTGTCTCGTGAAGATTAC  300
TATTTCCACAAAGATGCATCTACCTTCCGTCCGGAAGTTTTCAAGAAGTT  350
CATGTCCCACTTTAAAGGCAAGGAATATGTTACTCTGGAAGACGCAGCTA  400
GCGCCCGTTATGCAATGGTACAGGAAAGCCGCAAAAAAAACCCGACTTTC  450
ACTTACACCGTTCAGCAGCGTATCACCAGTTACGGTGAAACGATTAAATA  500
TTTCCGTACCATTGTTGAACCGGCTACTGGCAAGTGCCCGGTTGCGTGGA  550
TTAAGATCCTGTTTGAACAGGAGCGACTGCCGTACAACGAGGGTTGGCGT  600
CCGCCTAAAGCTGAACTGTCTGGCTTTAGTATGGCATCCGATGTCCTGGA  650
GCTGGCGTTAGTGACCCCGGAAAAACTGATCGACAAACCGTGTGAAGGCA  700
AACAGTGCCCGCAAGCCCGCGGCATCCACGGTTACTTCGGCATGCTGCTG  750
CCGATCACTGCGCAGGAACTGGCAGTTAAGTAA 783
```

## B

```
ATGGGCAGCAGCCATCATCATCATCATCACAGCAGCGGCCTGGTGCCGCG   50
CGGCAGCCATATGGCACCGTGGAAAGCTCCTGGTCCGGACGACGTTCGCG  100
GTCCGTGTCCAATGCTGAATACACTGGCTAACCACGGTTTCCTGCCGCAC  150
GACGGTAAAAACATTGACGTTAATACCACCGTGAACGCTCTGTCCTCCGC  200
GCTGAACCTGGACGACGAATTATCCCGCGATCTGCACACCTTCGCAGTGA  250
CGACCAACCCGCAGCCGAACGCTACGTGGTTTAGCCTGAACCACTTATCC  300
CGCCACAATGTGCTGGAACACGATGCATCTCTGTCCCGCCAGGATGCGTA  350
TTTCGGTCCACCTGATGTTTTCAACGCGGCTGTGTTCAATGAAACCAAGG  400
CTTATTGGACAGGCGACATCATTAACTTCCAGATGGCTGCGAACGCGTTG  450
ACCGCGCGTCTGATGACCTCCAACCTGACTAACCCGGAATTCTCTATGTC  500
CCAGCTGGGTCGTGGCTTCGGTCTGGGCGAAACTGTTGCTTATGTAACTA  550
TCCTGGGCTCTAAAGAAACACGCACTGTACCGAAGGCGTTTGTTGAATAC  600
CTGTTCGAAAACGAACGTCTGCCGTACGAACTGGGTTTTAAAAAGATGAA  650
ATCTGCTCTGACTGAAGATGAACTGACTACCATGATGGGTGAAATTTATT  700
CTCTGCAACACCTGCCGGAAAGCTTTACCAAACCGTTCGCAAAACGTAGC  750
GAAGCGCCGTTCGAAAAACGTGCGGAAAAACGCTGCCCGTTCCACTAA  798
```

**FIG S1.** Optimized nucleotide sequences used for *E. coli* expression of the *C. virescens* (**A**) and *D. caldariorum* (**B**) UPOs.

## A

M*ELDFSKWKTRQPGEFRAPCPAMNSLANHGFIPRDGRNITVAMLVPVLQE* 50 *(49)*
*VFHLSPELAQTISTLGLFTAQDPSKGVFTLDDLNRHNLFEHDASLSREDY* 100 *(99)*
*YFHKDASTFRPEVFKKFMSHFKGKEYVTLEDAASARYAMVQESRKKNPTF* 150 *(149)*
*TYTVQQRITSYGETIKYFRTIVEPATGKCPVAWIKILFEQERLPYNEGWR* 200 *(199)*
*PPKAELSGFSMASDVLELALVTPEKLIDKPCEGKQCPQARGIHGYFGMLL* 250 *(249)*
*PITAQELAVK* 260 *(259)*

- Total protein: 260 amino acids, with theoretical pI/Mw of 7.69/29617 Da
- Mature protein: *259* amino acids (*italics*)
- Trypsin hydrolysis giving the following 12 peptides (176 aa, 68%): A18-R34, G76-R85, H86-K104, D105-K116, G123-R136, Y137-R144, K145-R157, I158-R169, T170-K185, I186-K203, A204-K225 and L226-R240 (numbering of peptide residues corresponds to the complete sequence indicated above)

## B

MGSSHHHHHHSSGLVPRGSHM*APWKAPGPDDVRGPCPMLNTLANHGFLPH* 50 *(29)*
*DGKNIDVNTTVNALSSALNLDDELSRDLHTFAVTTNPQPNATWFSLNHLS* 100 *(79)*
*RHNVLEHDASLSRQDAYFGPPDVFNAAVFNETKAYWTGDIINFQMAANAL* 150 *(129)*
*TARLMTSNLTNPEFSMSQLGRGFGLGETVAYVTILGSKETRTVPKAFVEY* 200 *(179)*
*LFENERLPYELGFKKMKSALTEDELTTMMGEIYSLQHLPESFTKPFAKRS* 250 *(229)*
*EAPFEKRAEKRCPFH* 265 *(244)*

- Total protein: 265 amino acids, including poly-H tail and thrombin recognition sequence (underlined), with theoretical pI/Mw of 6.30/29609 Da
- Mature protein: *244* amino acids *(italics)*
- Trypsin hydrolysis giving the following 10 peptides (175 aa, 72%): G34-K53, N54-R76, D77-R101, H102-R113, Q114-K133, A134-R153, L154-R171, G172-K188, A196-K214 and L207-K215 (numbering of peptide residues corresponds to the complete sequence indicated above)

**FIG S2.** Amino-acid sequences of the *C. virescens* (A) and *D. caldariorum* (B) UPOs. Mature proteins are shown (residues and numbering in italics) with an initial methionine residue in the *C. virescens* UPO and additional poly-His sequence and contiguous residues (thrombin-recognition sequence included) in the *D. caldariorum* UPO. The predicted tryptic peptides were identified and their sequences (in blue/green or purple when overlapping) confirmed by LC-MS/MS. N-terminal sequences (in red) were determined by Edman degradation. Summaries of residue numbers and tryptic peptide composition are included below each sequence.

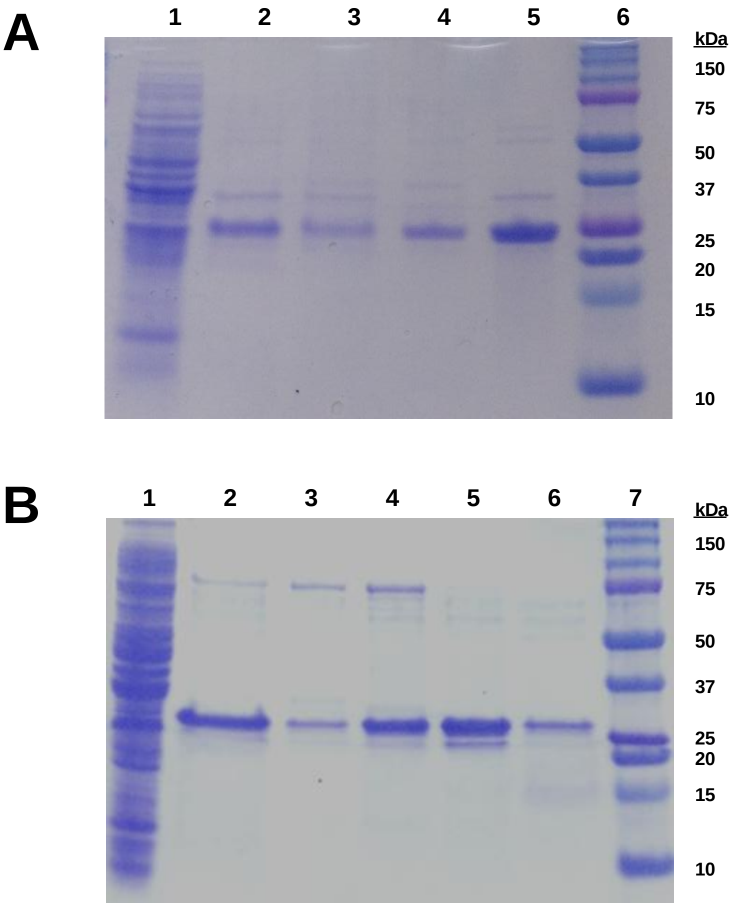


**FIG S3.** SDS-PAGE of samples collected during purification (**Fig. S4**) of the *C. virescens* (**A**) and *D. caldariorum* (**B**) UPOs produced in *E. coli*. The different lanes correspond to the initial cell lysates (*lane 1* in **A** and **B**), the subsequent ion-exchange (*lane 2* in **A**) or affinity (*lane 2* in **B**) chromatographies, and fractions from the final Superdex 75 chromatography (*lanes 3-5* in **A** and *lanes 3-6* in **B**, corresponding to fractions I-III and I-IV, respectively, in **Fig. S4B** and **D**) revealing nearly pure proteins of around 25 kDa. Molecular mass standards are included (*lanes 6* and *7* in **A** and **B**, respectively).

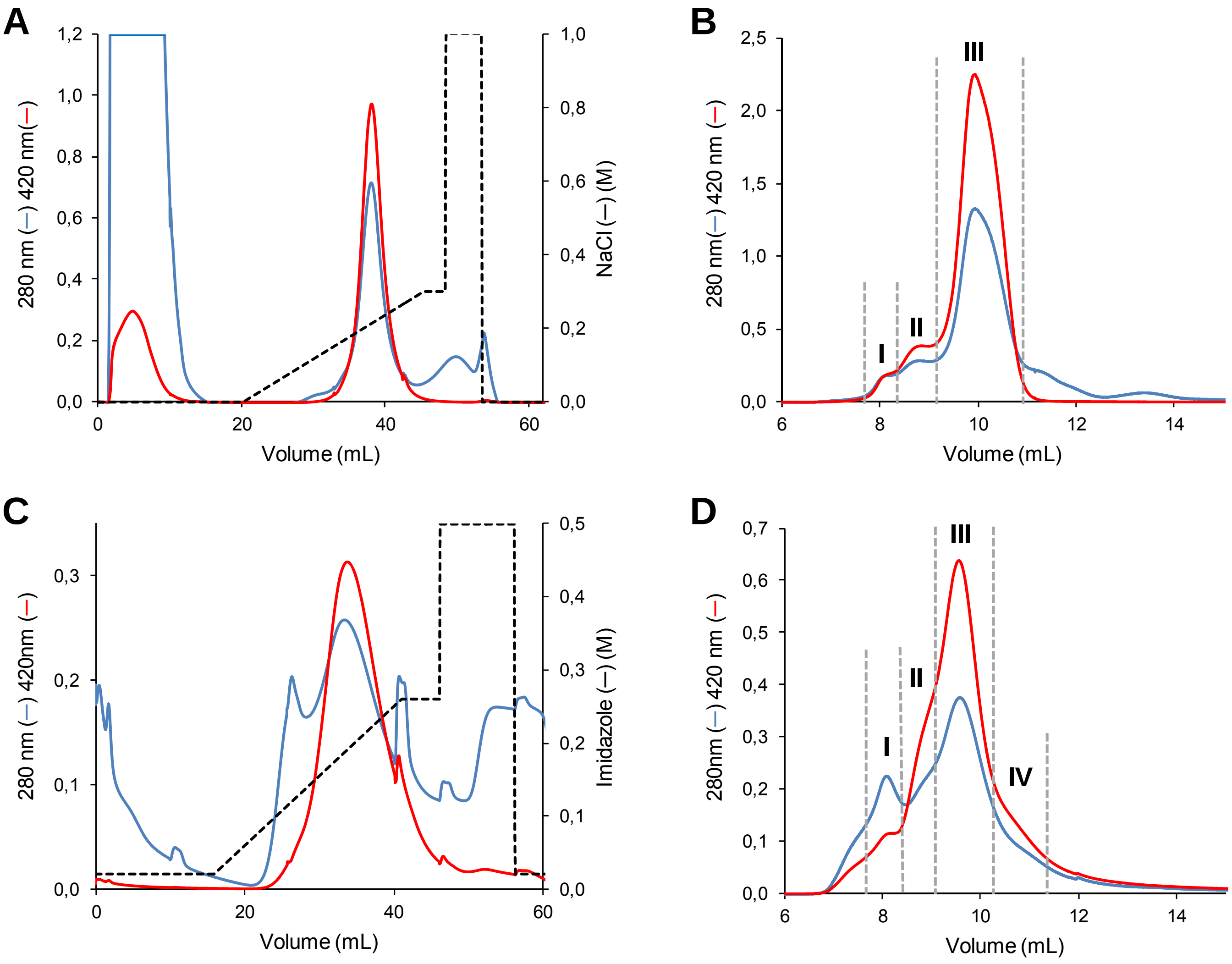


**FIG S4.** Main steps in the purification of the *C. virescens* (**A** and **B**) and *D. caldariorum* (**C** and **D**) UPOs from *E. coli* cultures, corresponding to cation exchange (**A**) or immobilized metal ion (**C**) chromatography, followed by Superdex 75 chromatography (**B** and **D**). The absorbance profiles at 280 nm (blue lines) and 420 nm (red lines) are shown, together with the NaCl and imidazole gradients (dashed lines) in **A** and **C**, respectively. Vertical dashed lines separate the different fractions collected in **B** and **D**, whose SDS-PAGE analyses are shown in **Fig. S3A** (*lanes 3-5*) and **B** (*lanes 3-6*), respectively.

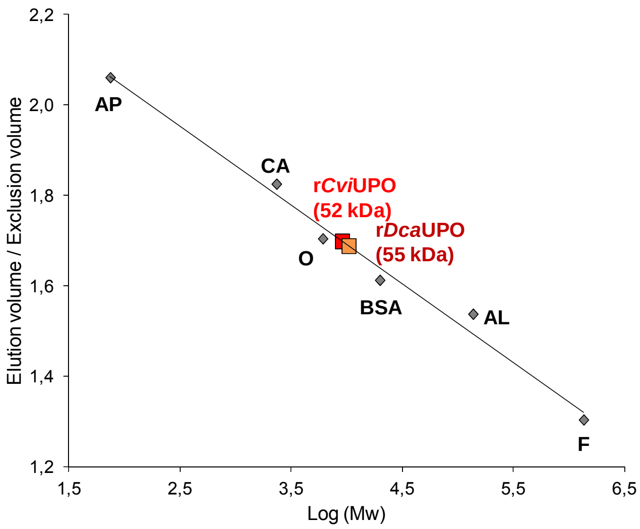


**FIG S5.** Calibration of Sepharose 12 10/300 column –using ferritin (F, 460 kDa), aldolase (AL, 170 kDa), bovine serum albumin (BSA, 73.5 kDa), ovoalbumin (O, 44.0 kDa), carbonic anhydrase (CA, 29.3 kDa) and aprotinin (AP, 6.5 kDa) as standars– for estimation of the native molecular masses of the recombinant *C. virescens* (*rCvi*UPO) and *D. caldariorum* (r*Dca*UPO) UPOs.

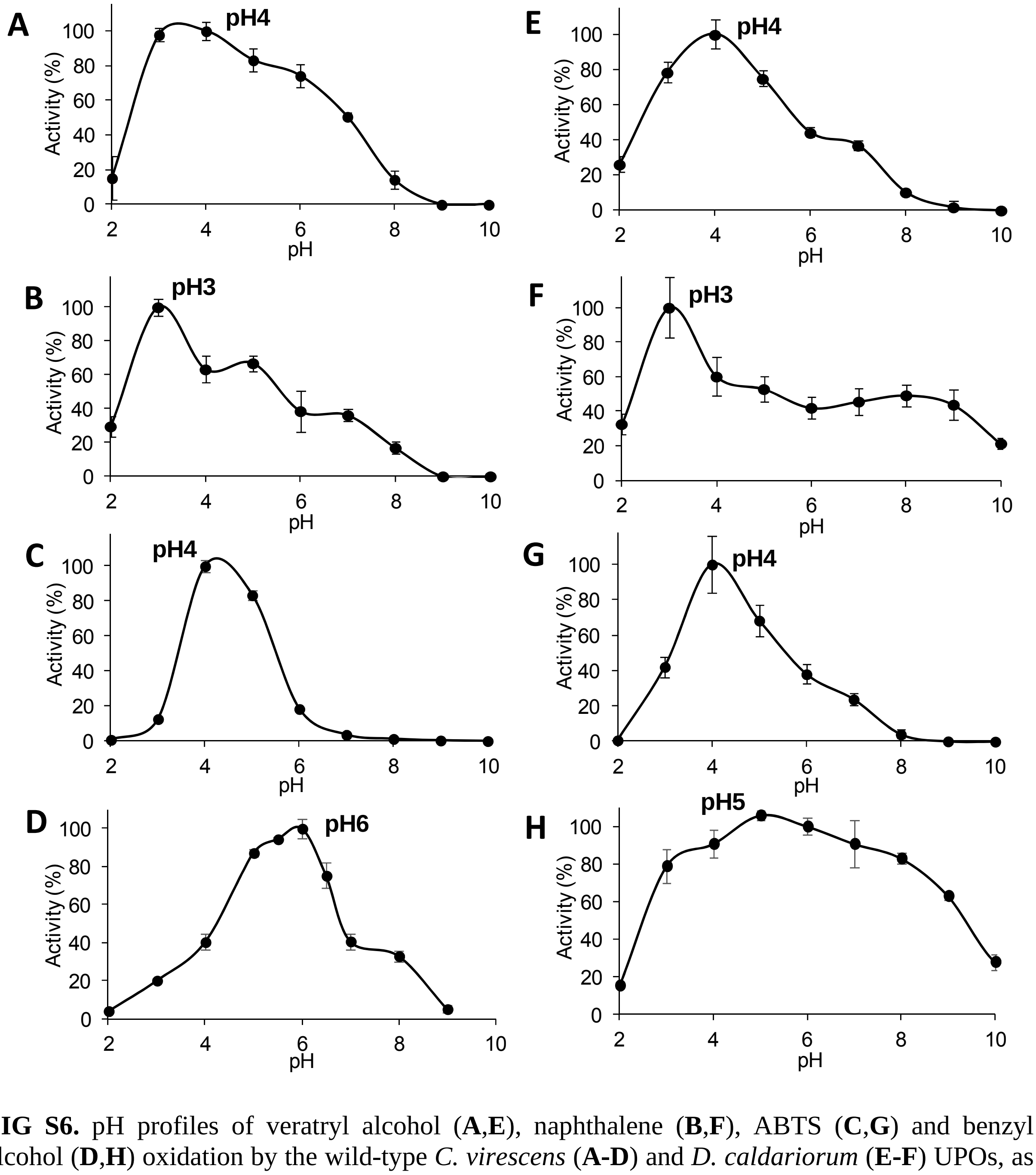


**FIG S6.** pH profiles of veratryl alcohol (**A**,**E**), naphthalene (**B**,**F**), ABTS (**C**,**G**) and benzyl alcohol (**D**,**H**) oxidation by the wild-type *C. virescens* (**A-D**) and *D. caldariorum* (**E-F**) UPOs, as relative activities referred to the optimal pH in each case (this optimum was used for kinetic estimations in **Table 2**). See Materials and Methods for details.

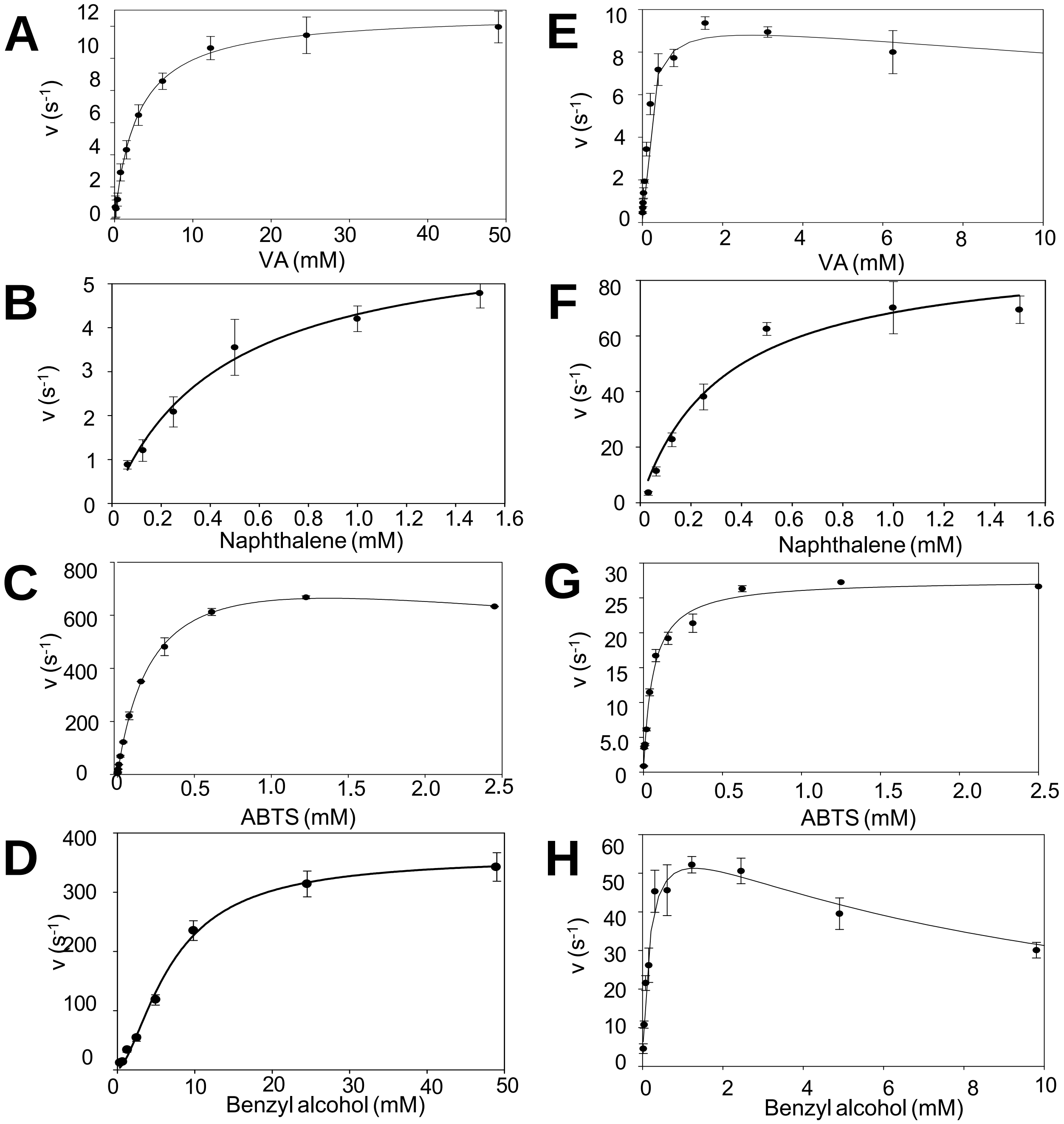


**FIG S7.** Kinetic curves of veratryl alcohol (**A** and **E**), ABTS (**B** and **F**), naphthalene (**C** and **G**) and benzyl alcohol oxidation by the wild-type *C. virescens* (**A-D**) and *D. caldariorum* (**E-H**) UPOs, fitted to equations 1-3 that yielded the kinetic constants shown in **Table 2**.

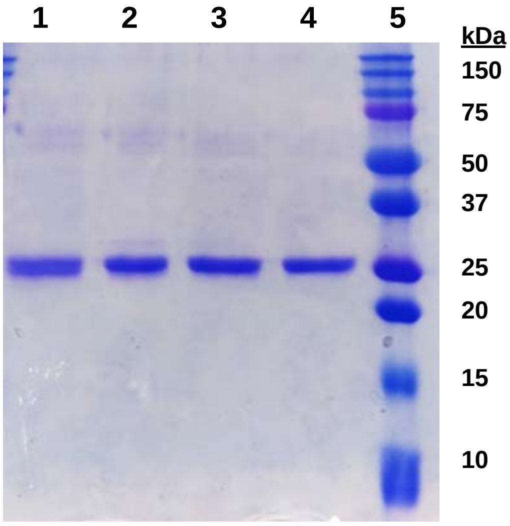


**FIG S8.** SDS-PAGE of purified *D. caldariorum* UPO (*lane 1*), *C. virescens* UPO (*lane 2*), and its F88L (*lane 3*) and T158F (*lane 4*) variants with molecular masses around 25 kDa. Molecular-mass standards are included in *lane 5*.

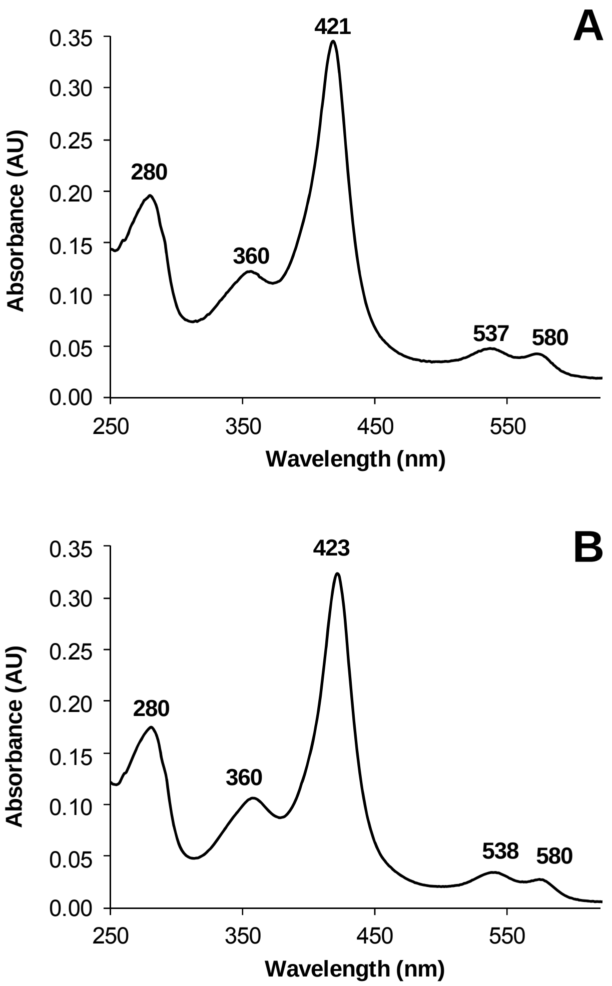


**FIG 9.** UV-vis spectra of the F88L (**A**) and T158F (**B**) variants of the *C. virescens* UPO.

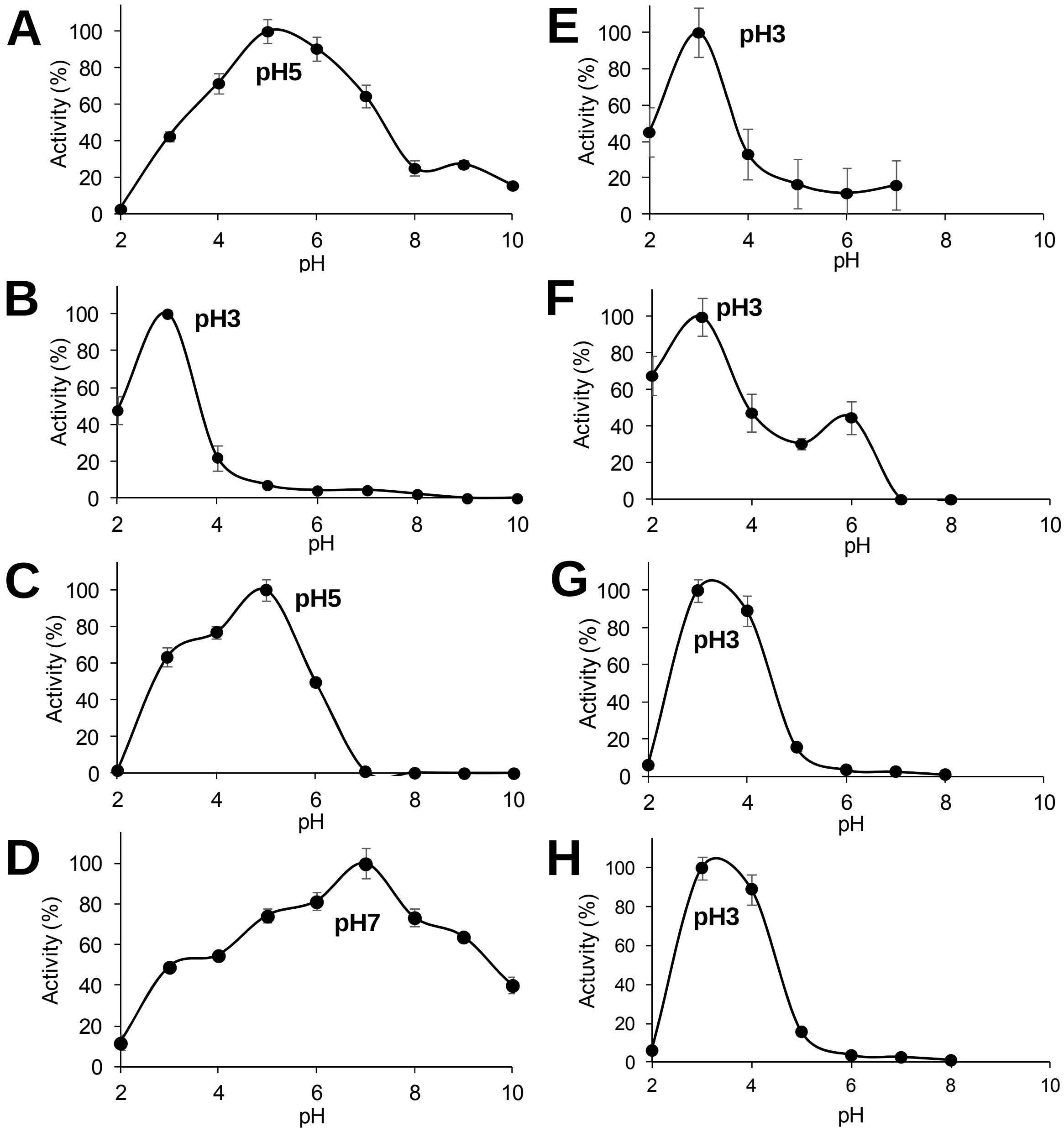


**FIG S10.** pH profiles of veratryl alcohol (**A** and **E**), naphthalene (**B** and **F**), ABTS (**C** and **G**) and benzyl alcohol (**D** and **H**) oxidation by the F88L (**A-D**) and T158F (**E-H**) variants of *C. virescens* UPOs, as relative activities referred to the optimal pH in each case (this optimum was used for kinetic estimations in **Table 3**). See Materials and Methods for details.

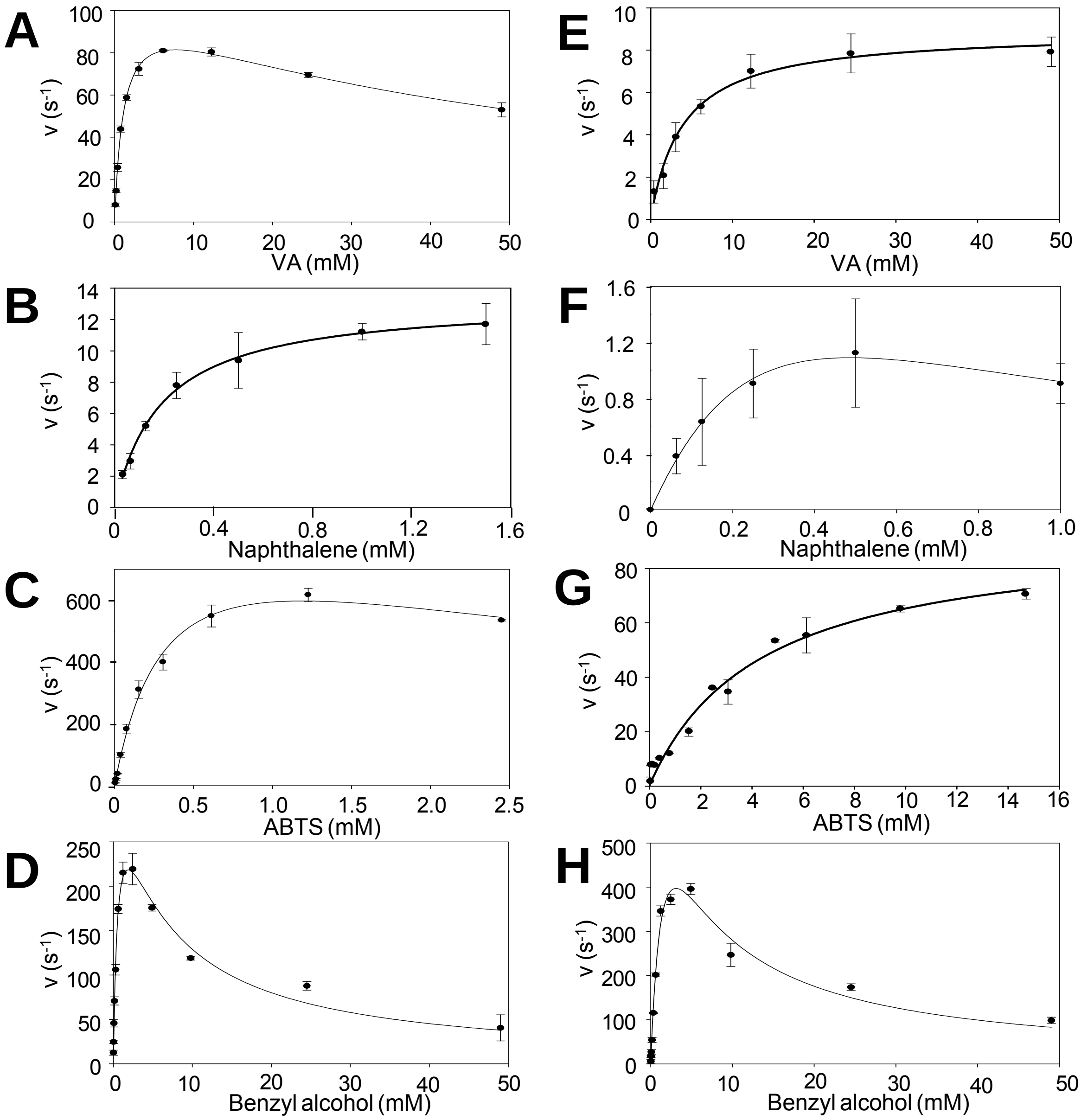


**FIG S11.** Kinetic curves of veratryl alcohol (**A** and **E**), naphthalene (**B** and **F**), ABTS (**C** and **G**) and benzyl alcohol (**D** and **H**) oxidation by the F88L (**A-D**) and T158F (**E-H**) variants of *C. virescens* UPO, which were fitted to Eqs. 1 (**B, E** and **G**) and 2 (**A, C, D, F** and **H**) yielding the kinetic constants shown in **Table 3**.
